# Supplementary material for: Increasing in vivo drug exposure levels of compound WX-081 (sudapyridine) when used in combination with clofazimine or clarithromycin
Source: Microbiol Spectr. 2026 Feb 26;14(4):e01555-25. doi: 10.1128/spectrum.01555-25 (PMC13055377; doi:10.1128/spectrum.01555-25)
Supplement: Supplemental material — Fig. S1 and S2; Tables S1 and S2. [file spectrum.01555-25-s0001.docx]

**Supplementary**

**Supplementary Material 1**


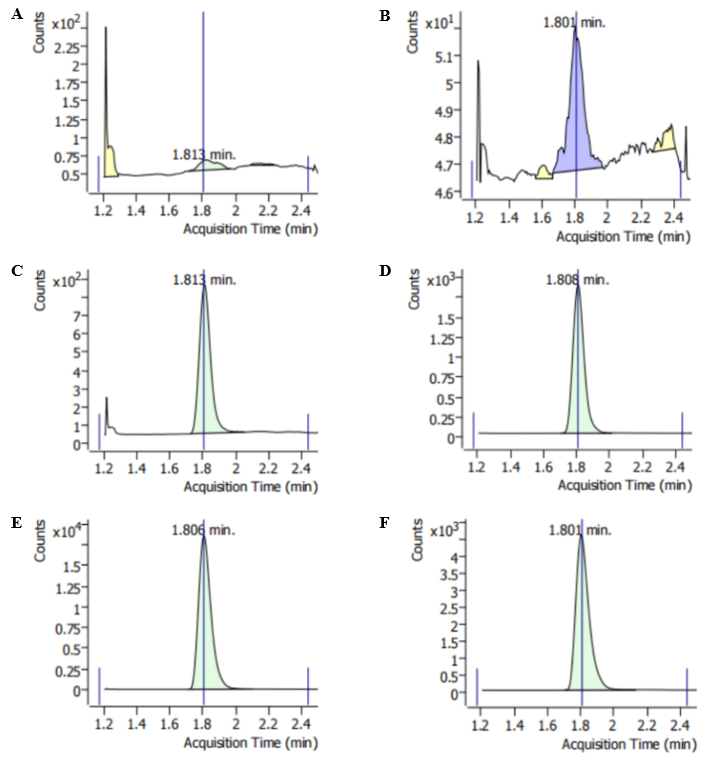


**Figure S1.** Typical LC-MS/MS chromatograms of WX-081 and WX-081-M3 blank matrices (A, B, respectively); Chromatograms of WX-081 and WX-081-M3 standard samples (C, D, respectively); Chromatograms of WX-081 and WX-081-M3 plasma samples to be tested (E, F, respectively).


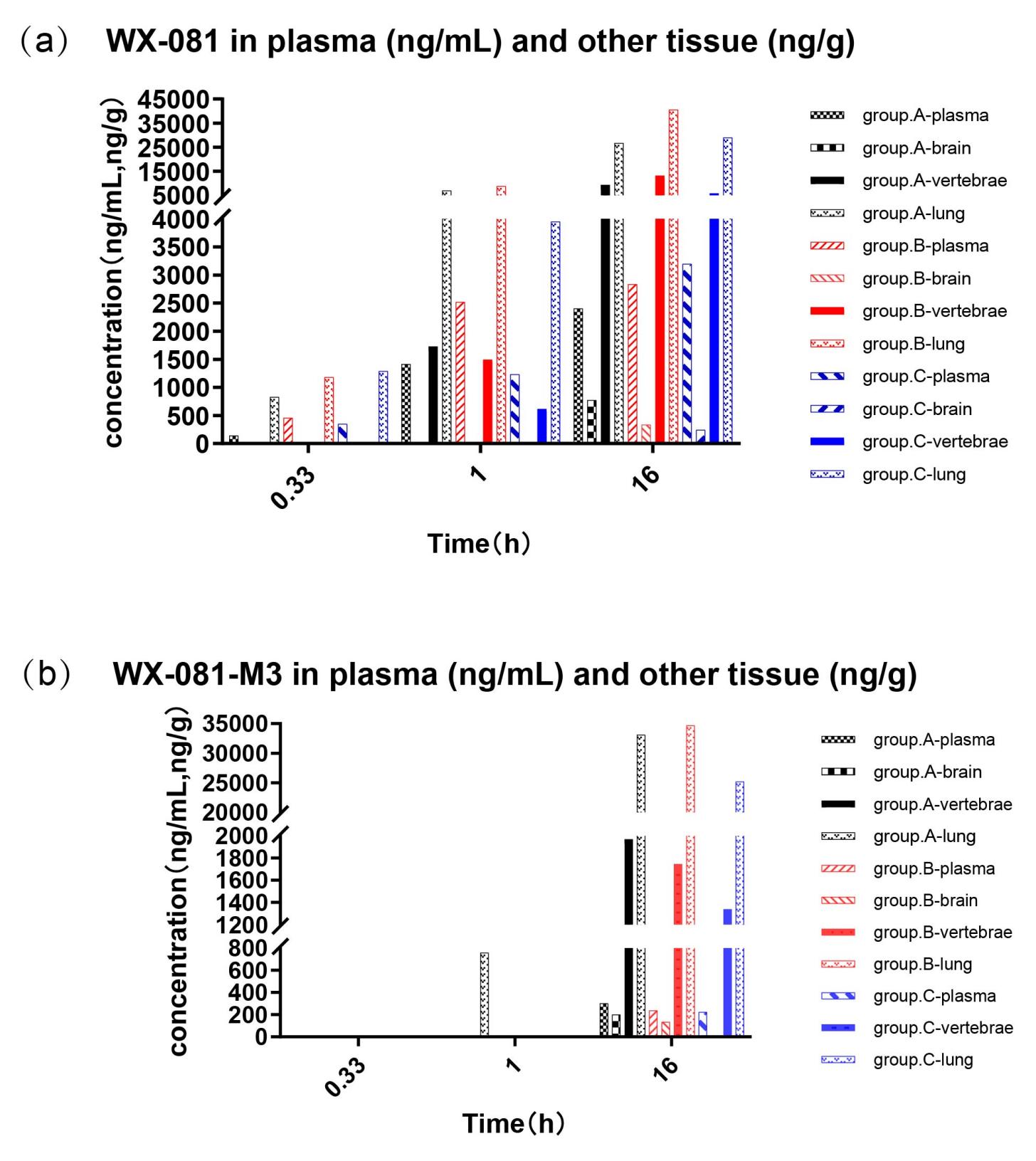


**Figure S2.** The concentration of WX-081(a), WX-081-M3(b) in plasma (ng/mL) and other tissue (ng/g).

**Supplementary Material 2**

**Table S1.** Concentration determination and accuracy analysis of WX-081 and WX-081-M3 in plasma, brain, vertebrae and lung tissue.

| matrix | Theoretical concentration(ng/mL) | WX-081 | | WX-081-M3 | |
| --- | --- | --- | --- | --- | --- |
|  |  | Actual concentration  (ng/mL) | Accuracy(%) | Actual concentration  (ng/mL) | Accuracy(%) |
| plasma | 100 | 122.8 | 122.8 | 140.7 | 140.7 |
|  | 500 | 524.1 | 104.8 | 536.6 | 107.3 |
|  | 800 | 780.2 | 97.5 | 796.4 | 99.5 |
| brain tissue | 20 | 21.9 | 109.6 | 31.2 | 155.9 |
|  | 500 | 509.9 | 102.0 | 481.0 | 96.2 |
|  | 800 | 673.9 | 84.2 | 601.2 | 75.1 |
| vertebrae | 100 | 111.8 | 111.8 | 106.2 | 106.2 |
|  | 500 | 629.9 | 126.0 | 638.4 | 127.7 |
|  | 800 | 889.3 | 111.2 | 866.7 | 108.3 |
| lung tissue | 100 | 112.9 | 112.9 | 109.8 | 109.8 |
|  | 500 | 554.4 | 110.9 | 556.2 | 111.2 |
|  | 800 | 864.2 | 108 | 851.8 | 106.5 |

**Supplementary Material 3**

**Table S2.** Standard curves, linear ranges and Pearson correlation coefficient (r) of WX-081 and WX-081-M3 in plasma and other tissues.

| compound | matrix | standard curve | Linear range(ng/mL) | r |
| --- | --- | --- | --- | --- |
| WX-081 | plasma | y = 0.009703 * x + 0.060795 | 50-1000 | 0.995 |
|  |  | y = 0.013535 * x + 0.051278 | 50-1000 | 0.992 |
|  | brain tissue | y = 0.015561 * x + 0.067074 | 10-1000 | 0.998 |
|  | vertebrae | y = 0.004533 * x + 0.091668 | 50-1000 | 0.991 |
|  | lung tissue | y = 0.013037 * x + 0.079991 | 50-1000 | 0.996 |
|  |  | y = 0.012516 * x + 0.103687 | 50-1000 | 0.990 |
| WX-081-M3 | plasma | y = 0.018647 * x + 0.173002 | 50-1000 | 0.990 |
|  |  | y = 0.010495 * x + 0.173623 | 50-1000 | 0.992 |
|  | brain tissue | y = 0.014404 * x + 0.031028 | 10-1000 | 0.990 |
|  | vertebrae | y = 0.003473 * x + 0.122812 | 50-1000 | 0.992 |
|  | lung tissue | y = 0.011584 * x + 0.144044 | 50-1000 | 0.990 |
|  |  | y = 0.010427 * x + 0.136313 | 50-1000 | 0.990 |

[1] Cowman, S., van Ingen J., Griffith D. E., et al. Non-tuberculous mycobacterial pulmonary disease [J]. Eur Respir J, 2019, 54(1).

[2] Huang, Z., Luo W., Xu D., et al. Discovery and preclinical profile of sudapyridine (WX-081), a novel anti-tuberculosis agent [J]. Bioorg Med Chem Lett, 2022, 71: 128824.

[1] Cowman, S., van Ingen J., Griffith D. E., et al. Non-tuberculous mycobacterial pulmonary disease [J]. Eur Respir J, 2019, 54(1).

[2] Dahl, V. N., Molhave M., Floe A., et al. Global trends of pulmonary infections with nontuberculous mycobacteria: a systematic review [J]. Int J Infect Dis, 2022, 125: 120-31.

[1] Cowman, S., van Ingen J., Griffith D. E., et al. Non-tuberculous mycobacterial pulmonary disease [J]. Eur Respir J, 2019, 54(1).

[2] Dahl, V. N., Molhave M., Floe A., et al. Global trends of pulmonary infections with nontuberculous mycobacteria: a systematic review [J]. Int J Infect Dis, 2022, 125: 120-31.

[3] Griffith, D. E., Daley C. L. Treatment of Mycobacterium abscessus Pulmonary Disease [J]. Chest, 2022, 161(1): 64-75.

[1] Cowman, S., van Ingen J., Griffith D. E., et al. Non-tuberculous mycobacterial pulmonary disease [J]. Eur Respir J, 2019, 54(1).

[2] Dahl, V. N., Molhave M., Floe A., et al. Global trends of pulmonary infections with nontuberculous mycobacteria: a systematic review [J]. Int J Infect Dis, 2022, 125: 120-31.

[3] Griffith, D. E., Daley C. L. Treatment of Mycobacterium abscessus Pulmonary Disease [J]. Chest, 2022, 161(1): 64-75.

[4] van der Laan, R., Snabilie A., Obradovic M. Meeting the challenges of NTM-PD from the perspective of the organism and the disease process: innovations in drug development and delivery [J]. Respir Res, 2022, 23(1): 376.

[2] Dahl, V. N., Molhave M., Floe A., et al. Global trends of pulmonary infections with nontuberculous mycobacteria: a systematic review [J]. Int J Infect Dis, 2022, 125: 120-31.

[3] Griffith, D. E., Daley C. L. Treatment of Mycobacterium abscessus Pulmonary Disease [J]. Chest, 2022, 161(1): 64-75.

[4] van der Laan, R., Snabilie A., Obradovic M. Meeting the challenges of NTM-PD from the perspective of the organism and the disease process: innovations in drug development and delivery [J]. Respir Res, 2022, 23(1): 376.

[5] Daley, C. L., Iaccarino J. M., Lange C., et al. Treatment of Nontuberculous Mycobacterial Pulmonary Disease: An Official ATS/ERS/ESCMID/IDSA Clinical Practice Guideline [J]. Clin Infect Dis, 2020, 71(4): 905-13.

[1] Cowman, S., van Ingen J., Griffith D. E., et al. Non-tuberculous mycobacterial pulmonary disease [J]. Eur Respir J, 2019, 54(1).

[2] Dahl, V. N., Molhave M., Floe A., et al. Global trends of pulmonary infections with nontuberculous mycobacteria: a systematic review [J]. Int J Infect Dis, 2022, 125: 120-31.

[3] Griffith, D. E., Daley C. L. Treatment of Mycobacterium abscessus Pulmonary Disease [J]. Chest, 2022, 161(1): 64-75.

[4] van der Laan, R., Snabilie A., Obradovic M. Meeting the challenges of NTM-PD from the perspective of the organism and the disease process: innovations in drug development and delivery [J]. Respir Res, 2022, 23(1): 376.

[5] Daley, C. L., Iaccarino J. M., Lange C., et al. Treatment of Nontuberculous Mycobacterial Pulmonary Disease: An Official ATS/ERS/ESCMID/IDSA Clinical Practice Guideline [J]. Clin Infect Dis, 2020, 71(4): 905-13.

[6] Rapid communication: key changes to treatment of multidrug- and rifampicin-resistant tuberculosis (MDR/RR-TB). [Z]. Geneva; World Health Organization. 2018

[1] Cowman, S., van Ingen J., Griffith D. E., et al. Non-tuberculous mycobacterial pulmonary disease [J]. Eur Respir J, 2019, 54(1).

[2] Dahl, V. N., Molhave M., Floe A., et al. Global trends of pulmonary infections with nontuberculous mycobacteria: a systematic review [J]. Int J Infect Dis, 2022, 125: 120-31.

[3] Griffith, D. E., Daley C. L. Treatment of Mycobacterium abscessus Pulmonary Disease [J]. Chest, 2022, 161(1): 64-75.

[4] van der Laan, R., Snabilie A., Obradovic M. Meeting the challenges of NTM-PD from the perspective of the organism and the disease process: innovations in drug development and delivery [J]. Respir Res, 2022, 23(1): 376.

[5] Daley, C. L., Iaccarino J. M., Lange C., et al. Treatment of Nontuberculous Mycobacterial Pulmonary Disease: An Official ATS/ERS/ESCMID/IDSA Clinical Practice Guideline [J]. Clin Infect Dis, 2020, 71(4): 905-13.

[6] Rapid communication: key changes to treatment of multidrug- and rifampicin-resistant tuberculosis (MDR/RR-TB). [Z]. Geneva; World Health Organization. 2018

[1] Cowman, S., van Ingen J., Griffith D. E., et al. Non-tuberculous mycobacterial pulmonary disease [J]. Eur Respir J, 2019, 54(1).

[2] Dahl, V. N., Molhave M., Floe A., et al. Global trends of pulmonary infections with nontuberculous mycobacteria: a systematic review [J]. Int J Infect Dis, 2022, 125: 120-31.

[3] Griffith, D. E., Daley C. L. Treatment of Mycobacterium abscessus Pulmonary Disease [J]. Chest, 2022, 161(1): 64-75.

[4] van der Laan, R., Snabilie A., Obradovic M. Meeting the challenges of NTM-PD from the perspective of the organism and the disease process: innovations in drug development and delivery [J]. Respir Res, 2022, 23(1): 376.

[5] Daley, C. L., Iaccarino J. M., Lange C., et al. Treatment of Nontuberculous Mycobacterial Pulmonary Disease: An Official ATS/ERS/ESCMID/IDSA Clinical Practice Guideline [J]. Clin Infect Dis, 2020, 71(4): 905-13.

[6] Rapid communication: key changes to treatment of multidrug- and rifampicin-resistant tuberculosis (MDR/RR-TB). [Z]. Geneva; World Health Organization. 2018

[1] Cowman, S., van Ingen J., Griffith D. E., et al. Non-tuberculous mycobacterial pulmonary disease [J]. Eur Respir J, 2019, 54(1).

[2] Dahl, V. N., Molhave M., Floe A., et al. Global trends of pulmonary infections with nontuberculous mycobacteria: a systematic review [J]. Int J Infect Dis, 2022, 125: 120-31.

[3] Griffith, D. E., Daley C. L. Treatment of Mycobacterium abscessus Pulmonary Disease [J]. Chest, 2022, 161(1): 64-75.

[4] van der Laan, R., Snabilie A., Obradovic M. Meeting the challenges of NTM-PD from the perspective of the organism and the disease process: innovations in drug development and delivery [J]. Respir Res, 2022, 23(1): 376.

[5] Daley, C. L., Iaccarino J. M., Lange C., et al. Treatment of Nontuberculous Mycobacterial Pulmonary Disease: An Official ATS/ERS/ESCMID/IDSA Clinical Practice Guideline [J]. Clin Infect Dis, 2020, 71(4): 905-13.

[6] Rapid communication: key changes to treatment of multidrug- and rifampicin-resistant tuberculosis (MDR/RR-TB). [Z]. Geneva; World Health Organization. 2018

[7] Ruth, M. M., Sangen J. J. N., Remmers K., et al. A bedaquiline/clofazimine combination regimen might add activity to the treatment of clinically relevant non-tuberculous mycobacteria [J]. J Antimicrob Chemother, 2019, 74(4): 935-43.

[1] Cowman, S., van Ingen J., Griffith D. E., et al. Non-tuberculous mycobacterial pulmonary disease [J]. Eur Respir J, 2019, 54(1).

[2] Dahl, V. N., Molhave M., Floe A., et al. Global trends of pulmonary infections with nontuberculous mycobacteria: a systematic review [J]. Int J Infect Dis, 2022, 125: 120-31.

[3] Griffith, D. E., Daley C. L. Treatment of Mycobacterium abscessus Pulmonary Disease [J]. Chest, 2022, 161(1): 64-75.

[4] van der Laan, R., Snabilie A., Obradovic M. Meeting the challenges of NTM-PD from the perspective of the organism and the disease process: innovations in drug development and delivery [J]. Respir Res, 2022, 23(1): 376.

[5] Daley, C. L., Iaccarino J. M., Lange C., et al. Treatment of Nontuberculous Mycobacterial Pulmonary Disease: An Official ATS/ERS/ESCMID/IDSA Clinical Practice Guideline [J]. Clin Infect Dis, 2020, 71(4): 905-13.

[6] Rapid communication: key changes to treatment of multidrug- and rifampicin-resistant tuberculosis (MDR/RR-TB). [Z]. Geneva; World Health Organization. 2018

[7] Ruth, M. M., Sangen J. J. N., Remmers K., et al. A bedaquiline/clofazimine combination regimen might add activity to the treatment of clinically relevant non-tuberculous mycobacteria [J]. J Antimicrob Chemother, 2019, 74(4): 935-43.

[1] Cowman, S., van Ingen J., Griffith D. E., et al. Non-tuberculous mycobacterial pulmonary disease [J]. Eur Respir J, 2019, 54(1).

[2] Dahl, V. N., Molhave M., Floe A., et al. Global trends of pulmonary infections with nontuberculous mycobacteria: a systematic review [J]. Int J Infect Dis, 2022, 125: 120-31.

[3] Griffith, D. E., Daley C. L. Treatment of Mycobacterium abscessus Pulmonary Disease [J]. Chest, 2022, 161(1): 64-75.

[4] van der Laan, R., Snabilie A., Obradovic M. Meeting the challenges of NTM-PD from the perspective of the organism and the disease process: innovations in drug development and delivery [J]. Respir Res, 2022, 23(1): 376.

[5] Daley, C. L., Iaccarino J. M., Lange C., et al. Treatment of Nontuberculous Mycobacterial Pulmonary Disease: An Official ATS/ERS/ESCMID/IDSA Clinical Practice Guideline [J]. Clin Infect Dis, 2020, 71(4): 905-13.

[6] Rapid communication: key changes to treatment of multidrug- and rifampicin-resistant tuberculosis (MDR/RR-TB). [Z]. Geneva; World Health Organization. 2018

[7] Ruth, M. M., Sangen J. J. N., Remmers K., et al. A bedaquiline/clofazimine combination regimen might add activity to the treatment of clinically relevant non-tuberculous mycobacteria [J]. J Antimicrob Chemother, 2019, 74(4): 935-43.

[9] Pym, A. S., Diacon A. H., Tang S. J., et al. Bedaquiline in the treatment of multidrug- and extensively drug-resistant tuberculosis [J]. Eur Respir J, 2016, 47(2): 564-74.

[1] Cowman, S., van Ingen J., Griffith D. E., et al. Non-tuberculous mycobacterial pulmonary disease [J]. Eur Respir J, 2019, 54(1).

[2] Dahl, V. N., Molhave M., Floe A., et al. Global trends of pulmonary infections with nontuberculous mycobacteria: a systematic review [J]. Int J Infect Dis, 2022, 125: 120-31.

[3] Griffith, D. E., Daley C. L. Treatment of Mycobacterium abscessus Pulmonary Disease [J]. Chest, 2022, 161(1): 64-75.

[4] van der Laan, R., Snabilie A., Obradovic M. Meeting the challenges of NTM-PD from the perspective of the organism and the disease process: innovations in drug development and delivery [J]. Respir Res, 2022, 23(1): 376.

[5] Daley, C. L., Iaccarino J. M., Lange C., et al. Treatment of Nontuberculous Mycobacterial Pulmonary Disease: An Official ATS/ERS/ESCMID/IDSA Clinical Practice Guideline [J]. Clin Infect Dis, 2020, 71(4): 905-13.

[6] Rapid communication: key changes to treatment of multidrug- and rifampicin-resistant tuberculosis (MDR/RR-TB). [Z]. Geneva; World Health Organization. 2018

[7] Ruth, M. M., Sangen J. J. N., Remmers K., et al. A bedaquiline/clofazimine combination regimen might add activity to the treatment of clinically relevant non-tuberculous mycobacteria [J]. J Antimicrob Chemother, 2019, 74(4): 935-43.

[8]
[9] Pym, A. S., Diacon A. H., Tang S. J., et al. Bedaquiline in the treatment of multidrug- and extensively drug-resistant tuberculosis [J]. Eur Respir J, 2016, 47(2): 564-74.

[10] Zhang, S. J., Yang Y., Sun W. W., et al. Effectiveness and safety of bedaquiline-containing regimens for treatment on patients with refractory RR/MDR/XDR-tuberculosis: a retrospective cohort study in East China [J]. BMC Infect Dis, 2022, 22(1): 715.

[1] Cowman, S., van Ingen J., Griffith D. E., et al. Non-tuberculous mycobacterial pulmonary disease [J]. Eur Respir J, 2019, 54(1).

[2] Dahl, V. N., Molhave M., Floe A., et al. Global trends of pulmonary infections with nontuberculous mycobacteria: a systematic review [J]. Int J Infect Dis, 2022, 125: 120-31.

[3] Griffith, D. E., Daley C. L. Treatment of Mycobacterium abscessus Pulmonary Disease [J]. Chest, 2022, 161(1): 64-75.

[4] van der Laan, R., Snabilie A., Obradovic M. Meeting the challenges of NTM-PD from the perspective of the organism and the disease process: innovations in drug development and delivery [J]. Respir Res, 2022, 23(1): 376.

[5] Daley, C. L., Iaccarino J. M., Lange C., et al. Treatment of Nontuberculous Mycobacterial Pulmonary Disease: An Official ATS/ERS/ESCMID/IDSA Clinical Practice Guideline [J]. Clin Infect Dis, 2020, 71(4): 905-13.

[6] Rapid communication: key changes to treatment of multidrug- and rifampicin-resistant tuberculosis (MDR/RR-TB). [Z]. Geneva; World Health Organization. 2018

[7] Ruth, M. M., Sangen J. J. N., Remmers K., et al. A bedaquiline/clofazimine combination regimen might add activity to the treatment of clinically relevant non-tuberculous mycobacteria [J]. J Antimicrob Chemother, 2019, 74(4): 935-43.

[9] Pym, A. S., Diacon A. H., Tang S. J., et al. Bedaquiline in the treatment of multidrug- and extensively drug-resistant tuberculosis [J]. Eur Respir J, 2016, 47(2): 564-74.

[10] Zhang, S. J., Yang Y., Sun W. W., et al. Effectiveness and safety of bedaquiline-containing regimens for treatment on patients with refractory RR/MDR/XDR-tuberculosis: a retrospective cohort study in East China [J]. BMC Infect Dis, 2022, 22(1): 715.

[1] Cowman, S., van Ingen J., Griffith D. E., et al. Non-tuberculous mycobacterial pulmonary disease [J]. Eur Respir J, 2019, 54(1).

[2] Dahl, V. N., Molhave M., Floe A., et al. Global trends of pulmonary infections with nontuberculous mycobacteria: a systematic review [J]. Int J Infect Dis, 2022, 125: 120-31.

[3] Griffith, D. E., Daley C. L. Treatment of Mycobacterium abscessus Pulmonary Disease [J]. Chest, 2022, 161(1): 64-75.

[5] Daley, C. L., Iaccarino J. M., Lange C., et al. Treatment of Nontuberculous Mycobacterial Pulmonary Disease: An Official ATS/ERS/ESCMID/IDSA Clinical Practice Guideline [J]. Clin Infect Dis, 2020, 71(4): 905-13.

[6] Rapid communication: key changes to treatment of multidrug- and rifampicin-resistant tuberculosis (MDR/RR-TB). [Z]. Geneva; World Health Organization. 2018

[7] Ruth, M. M., Sangen J. J. N., Remmers K., et al. A bedaquiline/clofazimine combination regimen might add activity to the treatment of clinically relevant non-tuberculous mycobacteria [J]. J Antimicrob Chemother, 2019, 74(4): 935-43.

[9] Pym, A. S., Diacon A. H., Tang S. J., et al. Bedaquiline in the treatment of multidrug- and extensively drug-resistant tuberculosis [J]. Eur Respir J, 2016, 47(2): 564-74.

[10] Zhang, S. J., Yang Y., Sun W. W., et al. Effectiveness and safety of bedaquiline-containing regimens for treatment on patients with refractory RR/MDR/XDR-tuberculosis: a retrospective cohort study in East China [J]. BMC Infect Dis, 2022, 22(1): 715.

[11] Gao, M., Gao J., Xie L., et al. Early outcome and safety of bedaquiline-containing regimens for treatment of MDR- and XDR-TB in China: a multicentre study [J]. Clin Microbiol Infect, 2021, 27(4): 597-602.

[1] Cowman, S., van Ingen J., Griffith D. E., et al. Non-tuberculous mycobacterial pulmonary disease [J]. Eur Respir J, 2019, 54(1).

[2] Dahl, V. N., Molhave M., Floe A., et al. Global trends of pulmonary infections with nontuberculous mycobacteria: a systematic review [J]. Int J Infect Dis, 2022, 125: 120-31.

[3] Griffith, D. E., Daley C. L. Treatment of Mycobacterium abscessus Pulmonary Disease [J]. Chest, 2022, 161(1): 64-75.

[4] van der Laan, R., Snabilie A., Obradovic M. Meeting the challenges of NTM-PD from the perspective of the organism and the disease process: innovations in drug development and delivery [J]. Respir Res, 2022, 23(1): 376.

[5] Daley, C. L., Iaccarino J. M., Lange C., et al. Treatment of Nontuberculous Mycobacterial Pulmonary Disease: An Official ATS/ERS/ESCMID/IDSA Clinical Practice Guideline [J]. Clin Infect Dis, 2020, 71(4): 905-13.

[6] Rapid communication: key changes to treatment of multidrug- and rifampicin-resistant tuberculosis (MDR/RR-TB). [Z]. Geneva; World Health Organization. 2018

[7] Ruth, M. M., Sangen J. J. N., Remmers K., et al. A bedaquiline/clofazimine combination regimen might add activity to the treatment of clinically relevant non-tuberculous mycobacteria [J]. J Antimicrob Chemother, 2019, 74(4): 935-43.

[9] Pym, A. S., Diacon A. H., Tang S. J., et al. Bedaquiline in the treatment of multidrug- and extensively drug-resistant tuberculosis [J]. Eur Respir J, 2016, 47(2): 564-74.

[10] Zhang, S. J., Yang Y., Sun W. W., et al. Effectiveness and safety of bedaquiline-containing regimens for treatment on patients with refractory RR/MDR/XDR-tuberculosis: a retrospective cohort study in East China [J]. BMC Infect Dis, 2022, 22(1): 715.

[11] Gao, M., Gao J., Xie L., et al. Early outcome and safety of bedaquiline-containing regimens for treatment of MDR- and XDR-TB in China: a multicentre study [J]. Clin Microbiol Infect, 2021, 27(4): 597-602.

[12] Huang, Z., Luo W., Xu D., et al. Discovery and preclinical profile of sudapyridine (WX-081), a novel anti-tuberculosis agent [J]. Bioorg Med Chem Lett, 2022, 71: 128824.

[1] Cowman, S., van Ingen J., Griffith D. E., et al. Non-tuberculous mycobacterial pulmonary disease [J]. Eur Respir J, 2019, 54(1).

[2] Dahl, V. N., Molhave M., Floe A., et al. Global trends of pulmonary infections with nontuberculous mycobacteria: a systematic review [J]. Int J Infect Dis, 2022, 125: 120-31.

[3] Griffith, D. E., Daley C. L. Treatment of Mycobacterium abscessus Pulmonary Disease [J]. Chest, 2022, 161(1): 64-75.

[4] van der Laan, R., Snabilie A., Obradovic M. Meeting the challenges of NTM-PD from the perspective of the organism and the disease process: innovations in drug development and delivery [J]. Respir Res, 2022, 23(1): 376.

[5] Daley, C. L., Iaccarino J. M., Lange C., et al. Treatment of Nontuberculous Mycobacterial Pulmonary Disease: An Official ATS/ERS/ESCMID/IDSA Clinical Practice Guideline [J]. Clin Infect Dis, 2020, 71(4): 905-13.

[6] Rapid communication: key changes to treatment of multidrug- and rifampicin-resistant tuberculosis (MDR/RR-TB). [Z]. Geneva; World Health Organization. 2018

[7] Ruth, M. M., Sangen J. J. N., Remmers K., et al. A bedaquiline/clofazimine combination regimen might add activity to the treatment of clinically relevant non-tuberculous mycobacteria [J]. J Antimicrob Chemother, 2019, 74(4): 935-43.

[9] Pym, A. S., Diacon A. H., Tang S. J., et al. Bedaquiline in the treatment of multidrug- and extensively drug-resistant tuberculosis [J]. Eur Respir J, 2016, 47(2): 564-74.

[10] Zhang, S. J., Yang Y., Sun W. W., et al. Effectiveness and safety of bedaquiline-containing regimens for treatment on patients with refractory RR/MDR/XDR-tuberculosis: a retrospective cohort study in East China [J]. BMC Infect Dis, 2022, 22(1): 715.

[11] Gao, M., Gao J., Xie L., et al. Early outcome and safety of bedaquiline-containing regimens for treatment of MDR- and XDR-TB in China: a multicentre study [J]. Clin Microbiol Infect, 2021, 27(4): 597-602.

[12] Huang, Z., Luo W., Xu D., et al. Discovery and preclinical profile of sudapyridine (WX-081), a novel anti-tuberculosis agent [J]. Bioorg Med Chem Lett, 2022, 71: 128824.

[13] Yao, R., Wang B., Fu L., et al. Sudapyridine (WX-081), a Novel Compound against Mycobacterium tuberculosis [J]. Microbiol Spectr, 2022, 10(1): e0247721.

[1] Cowman, S., van Ingen J., Griffith D. E., et al. Non-tuberculous mycobacterial pulmonary disease [J]. Eur Respir J, 2019, 54(1).

[2] Dahl, V. N., Molhave M., Floe A., et al. Global trends of pulmonary infections with nontuberculous mycobacteria: a systematic review [J]. Int J Infect Dis, 2022, 125: 120-31.

[3] Griffith, D. E., Daley C. L. Treatment of Mycobacterium abscessus Pulmonary Disease [J]. Chest, 2022, 161(1): 64-75.

[4] van der Laan, R., Snabilie A., Obradovic M. Meeting the challenges of NTM-PD from the perspective of the organism and the disease process: innovations in drug development and delivery [J]. Respir Res, 2022, 23(1): 376.

[5] Daley, C. L., Iaccarino J. M., Lange C., et al. Treatment of Nontuberculous Mycobacterial Pulmonary Disease: An Official ATS/ERS/ESCMID/IDSA Clinical Practice Guideline [J]. Clin Infect Dis, 2020, 71(4): 905-13.

[6] Rapid communication: key changes to treatment of multidrug- and rifampicin-resistant tuberculosis (MDR/RR-TB). [Z]. Geneva; World Health Organization. 2018

[7] Ruth, M. M., Sangen J. J. N., Remmers K., et al. A bedaquiline/clofazimine combination regimen might add activity to the treatment of clinically relevant non-tuberculous mycobacteria [J]. J Antimicrob Chemother, 2019, 74(4): 935-43.

[9] Pym, A. S., Diacon A. H., Tang S. J., et al. Bedaquiline in the treatment of multidrug- and extensively drug-resistant tuberculosis [J]. Eur Respir J, 2016, 47(2): 564-74.

[10] Zhang, S. J., Yang Y., Sun W. W., et al. Effectiveness and safety of bedaquiline-containing regimens for treatment on patients with refractory RR/MDR/XDR-tuberculosis: a retrospective cohort study in East China [J]. BMC Infect Dis, 2022, 22(1): 715.

[11] Gao, M., Gao J., Xie L., et al. Early outcome and safety of bedaquiline-containing regimens for treatment of MDR- and XDR-TB in China: a multicentre study [J]. Clin Microbiol Infect, 2021, 27(4): 597-602.

[12] Huang, Z., Luo W., Xu D., et al. Discovery and preclinical profile of sudapyridine (WX-081), a novel anti-tuberculosis agent [J]. Bioorg Med Chem Lett, 2022, 71: 128824.

[13] Yao, R., Wang B., Fu L., et al. Sudapyridine (WX-081), a Novel Compound against Mycobacterium tuberculosis [J]. Microbiol Spectr, 2022, 10(1): e0247721.

[14] Zhu, R., Shang Y., Chen S., et al. In Vitro Activity of the Sudapyridine (WX-081) against Non-Tuberculous Mycobacteria Isolated in Beijing, China [J]. Microbiol Spectr, 2022, 10(6): e0137222.

[1] Cowman, S., van Ingen J., Griffith D. E., et al. Non-tuberculous mycobacterial pulmonary disease [J]. Eur Respir J, 2019, 54(1).

[2] Dahl, V. N., Molhave M., Floe A., et al. Global trends of pulmonary infections with nontuberculous mycobacteria: a systematic review [J]. Int J Infect Dis, 2022, 125: 120-31.

[3] Griffith, D. E., Daley C. L. Treatment of Mycobacterium abscessus Pulmonary Disease [J]. Chest, 2022, 161(1): 64-75.

[4] van der Laan, R., Snabilie A., Obradovic M. Meeting the challenges of NTM-PD from the perspective of the organism and the disease process: innovations in drug development and delivery [J]. Respir Res, 2022, 23(1): 376.

[5] Daley, C. L., Iaccarino J. M., Lange C., et al. Treatment of Nontuberculous Mycobacterial Pulmonary Disease: An Official ATS/ERS/ESCMID/IDSA Clinical Practice Guideline [J]. Clin Infect Dis, 2020, 71(4): 905-13.

[6] Rapid communication: key changes to treatment of multidrug- and rifampicin-resistant tuberculosis (MDR/RR-TB). [Z]. Geneva; World Health Organization. 2018

[7] Ruth, M. M., Sangen J. J. N., Remmers K., et al. A bedaquiline/clofazimine combination regimen might add activity to the treatment of clinically relevant non-tuberculous mycobacteria [J]. J Antimicrob Chemother, 2019, 74(4): 935-43.

[9] Pym, A. S., Diacon A. H., Tang S. J., et al. Bedaquiline in the treatment of multidrug- and extensively drug-resistant tuberculosis [J]. Eur Respir J, 2016, 47(2): 564-74.

[10] Zhang, S. J., Yang Y., Sun W. W., et al. Effectiveness and safety of bedaquiline-containing regimens for treatment on patients with refractory RR/MDR/XDR-tuberculosis: a retrospective cohort study in East China [J]. BMC Infect Dis, 2022, 22(1): 715.

[11] Gao, M., Gao J., Xie L., et al. Early outcome and safety of bedaquiline-containing regimens for treatment of MDR- and XDR-TB in China: a multicentre study [J]. Clin Microbiol Infect, 2021, 27(4): 597-602.

[12] Huang, Z., Luo W., Xu D., et al. Discovery and preclinical profile of sudapyridine (WX-081), a novel anti-tuberculosis agent [J]. Bioorg Med Chem Lett, 2022, 71: 128824.

[13] Yao, R., Wang B., Fu L., et al. Sudapyridine (WX-081), a Novel Compound against Mycobacterium tuberculosis [J]. Microbiol Spectr, 2022, 10(1): e0247721.

[14] Zhu, R., Shang Y., Chen S., et al. In Vitro Activity of the Sudapyridine (WX-081) against Non-Tuberculous Mycobacteria Isolated in Beijing, China [J]. Microbiol Spectr, 2022, 10(6): e0137222.

[1] Cowman, S., van Ingen J., Griffith D. E., et al. Non-tuberculous mycobacterial pulmonary disease [J]. Eur Respir J, 2019, 54(1).

[2] Dahl, V. N., Molhave M., Floe A., et al. Global trends of pulmonary infections with nontuberculous mycobacteria: a systematic review [J]. Int J Infect Dis, 2022, 125: 120-31.

[3] Griffith, D. E., Daley C. L. Treatment of Mycobacterium abscessus Pulmonary Disease [J]. Chest, 2022, 161(1): 64-75.

[4] van der Laan, R., Snabilie A., Obradovic M. Meeting the challenges of NTM-PD from the perspective of the organism and the disease process: innovations in drug development and delivery [J]. Respir Res, 2022, 23(1): 376.

[5] Daley, C. L., Iaccarino J. M., Lange C., et al. Treatment of Nontuberculous Mycobacterial Pulmonary Disease: An Official ATS/ERS/ESCMID/IDSA Clinical Practice Guideline [J]. Clin Infect Dis, 2020, 71(4): 905-13.

[6] Rapid communication: key changes to treatment of multidrug- and rifampicin-resistant tuberculosis (MDR/RR-TB). [Z]. Geneva; World Health Organization. 2018

[7] Ruth, M. M., Sangen J. J. N., Remmers K., et al. A bedaquiline/clofazimine combination regimen might add activity to the treatment of clinically relevant non-tuberculous mycobacteria [J]. J Antimicrob Chemother, 2019, 74(4): 935-43.

[9] Pym, A. S., Diacon A. H., Tang S. J., et al. Bedaquiline in the treatment of multidrug- and extensively drug-resistant tuberculosis [J]. Eur Respir J, 2016, 47(2): 564-74.

[10] Zhang, S. J., Yang Y., Sun W. W., et al. Effectiveness and safety of bedaquiline-containing regimens for treatment on patients with refractory RR/MDR/XDR-tuberculosis: a retrospective cohort study in East China [J]. BMC Infect Dis, 2022, 22(1): 715.

[11] Gao, M., Gao J., Xie L., et al. Early outcome and safety of bedaquiline-containing regimens for treatment of MDR- and XDR-TB in China: a multicentre study [J]. Clin Microbiol Infect, 2021, 27(4): 597-602.

[12] Huang, Z., Luo W., Xu D., et al. Discovery and preclinical profile of sudapyridine (WX-081), a novel anti-tuberculosis agent [J]. Bioorg Med Chem Lett, 2022, 71: 128824.

[13] Yao, R., Wang B., Fu L., et al. Sudapyridine (WX-081), a Novel Compound against Mycobacterium tuberculosis [J]. Microbiol Spectr, 2022, 10(1): e0247721.

[14] Zhu, R., Shang Y., Chen S., et al. In Vitro Activity of the Sudapyridine (WX-081) against Non-Tuberculous Mycobacteria Isolated in Beijing, China [J]. Microbiol Spectr, 2022, 10(6): e0137222.

[1] Cowman, S., van Ingen J., Griffith D. E., et al. Non-tuberculous mycobacterial pulmonary disease [J]. Eur Respir J, 2019, 54(1).

[2] Dahl, V. N., Molhave M., Floe A., et al. Global trends of pulmonary infections with nontuberculous mycobacteria: a systematic review [J]. Int J Infect Dis, 2022, 125: 120-31.

[3] Griffith, D. E., Daley C. L. Treatment of Mycobacterium abscessus Pulmonary Disease [J]. Chest, 2022, 161(1): 64-75.

[4] van der Laan, R., Snabilie A., Obradovic M. Meeting the challenges of NTM-PD from the perspective of the organism and the disease process: innovations in drug development and delivery [J]. Respir Res, 2022, 23(1): 376.

[5] Daley, C. L., Iaccarino J. M., Lange C., et al. Treatment of Nontuberculous Mycobacterial Pulmonary Disease: An Official ATS/ERS/ESCMID/IDSA Clinical Practice Guideline [J]. Clin Infect Dis, 2020, 71(4): 905-13.

[6] Rapid communication: key changes to treatment of multidrug- and rifampicin-resistant tuberculosis (MDR/RR-TB). [Z]. Geneva; World Health Organization. 2018

[7] Ruth, M. M., Sangen J. J. N., Remmers K., et al. A bedaquiline/clofazimine combination regimen might add activity to the treatment of clinically relevant non-tuberculous mycobacteria [J]. J Antimicrob Chemother, 2019, 74(4): 935-43.

[9] Pym, A. S., Diacon A. H., Tang S. J., et al. Bedaquiline in the treatment of multidrug- and extensively drug-resistant tuberculosis [J]. Eur Respir J, 2016, 47(2): 564-74.

[10] Zhang, S. J., Yang Y., Sun W. W., et al. Effectiveness and safety of bedaquiline-containing regimens for treatment on patients with refractory RR/MDR/XDR-tuberculosis: a retrospective cohort study in East China [J]. BMC Infect Dis, 2022, 22(1): 715.

[11] Gao, M., Gao J., Xie L., et al. Early outcome and safety of bedaquiline-containing regimens for treatment of MDR- and XDR-TB in China: a multicentre study [J]. Clin Microbiol Infect, 2021, 27(4): 597-602.

[12] Huang, Z., Luo W., Xu D., et al. Discovery and preclinical profile of sudapyridine (WX-081), a novel anti-tuberculosis agent [J]. Bioorg Med Chem Lett, 2022, 71: 128824.

[13] Yao, R., Wang B., Fu L., et al. Sudapyridine (WX-081), a Novel Compound against Mycobacterium tuberculosis [J]. Microbiol Spectr, 2022, 10(1): e0247721.

[14] Zhu, R., Shang Y., Chen S., et al. In Vitro Activity of the Sudapyridine (WX-081) against Non-Tuberculous Mycobacteria Isolated in Beijing, China [J]. Microbiol Spectr, 2022, 10(6): e0137222.

[15] Brown-Elliott, B. A., Philley J. V., Griffith D. E., et al. In Vitro Susceptibility Testing of Bedaquiline against Mycobacterium avium Complex [J]. Antimicrob Agents Chemother, 2017, 61(2).

[1] Cowman, S., van Ingen J., Griffith D. E., et al. Non-tuberculous mycobacterial pulmonary disease [J]. Eur Respir J, 2019, 54(1).

[2] Dahl, V. N., Molhave M., Floe A., et al. Global trends of pulmonary infections with nontuberculous mycobacteria: a systematic review [J]. Int J Infect Dis, 2022, 125: 120-31.

[3] Griffith, D. E., Daley C. L. Treatment of Mycobacterium abscessus Pulmonary Disease [J]. Chest, 2022, 161(1): 64-75.

[4] van der Laan, R., Snabilie A., Obradovic M. Meeting the challenges of NTM-PD from the perspective of the organism and the disease process: innovations in drug development and delivery [J]. Respir Res, 2022, 23(1): 376.

[5] Daley, C. L., Iaccarino J. M., Lange C., et al. Treatment of Nontuberculous Mycobacterial Pulmonary Disease: An Official ATS/ERS/ESCMID/IDSA Clinical Practice Guideline [J]. Clin Infect Dis, 2020, 71(4): 905-13.

[6] Rapid communication: key changes to treatment of multidrug- and rifampicin-resistant tuberculosis (MDR/RR-TB). [Z]. Geneva; World Health Organization. 2018

[7] Ruth, M. M., Sangen J. J. N., Remmers K., et al. A bedaquiline/clofazimine combination regimen might add activity to the treatment of clinically relevant non-tuberculous mycobacteria [J]. J Antimicrob Chemother, 2019, 74(4): 935-43.

[9] Pym, A. S., Diacon A. H., Tang S. J., et al. Bedaquiline in the treatment of multidrug- and extensively drug-resistant tuberculosis [J]. Eur Respir J, 2016, 47(2): 564-74.

[10] Zhang, S. J., Yang Y., Sun W. W., et al. Effectiveness and safety of bedaquiline-containing regimens for treatment on patients with refractory RR/MDR/XDR-tuberculosis: a retrospective cohort study in East China [J]. BMC Infect Dis, 2022, 22(1): 715.

[11] Gao, M., Gao J., Xie L., et al. Early outcome and safety of bedaquiline-containing regimens for treatment of MDR- and XDR-TB in China: a multicentre study [J]. Clin Microbiol Infect, 2021, 27(4): 597-602.

[12] Huang, Z., Luo W., Xu D., et al. Discovery and preclinical profile of sudapyridine (WX-081), a novel anti-tuberculosis agent [J]. Bioorg Med Chem Lett, 2022, 71: 128824.

[13] Yao, R., Wang B., Fu L., et al. Sudapyridine (WX-081), a Novel Compound against Mycobacterium tuberculosis [J]. Microbiol Spectr, 2022, 10(1): e0247721.

[14] Zhu, R., Shang Y., Chen S., et al. In Vitro Activity of the Sudapyridine (WX-081) against Non-Tuberculous Mycobacteria Isolated in Beijing, China [J]. Microbiol Spectr, 2022, 10(6): e0137222.

[15] Brown-Elliott, B. A., Philley J. V., Griffith D. E., et al. In Vitro Susceptibility Testing of Bedaquiline against Mycobacterium avium Complex [J]. Antimicrob Agents Chemother, 2017, 61(2).

[16] Kim, D. H., Jhun B. W., Moon S. M., et al. In Vitro Activity of Bedaquiline and Delamanid against Nontuberculous Mycobacteria, Including Macrolide-Resistant Clinical Isolates [J]. Antimicrob Agents Chemother, 2019, 63(8).

[1] Cowman, S., van Ingen J., Griffith D. E., et al. Non-tuberculous mycobacterial pulmonary disease [J]. Eur Respir J, 2019, 54(1).

[2] Dahl, V. N., Molhave M., Floe A., et al. Global trends of pulmonary infections with nontuberculous mycobacteria: a systematic review [J]. Int J Infect Dis, 2022, 125: 120-31.

[3] Griffith, D. E., Daley C. L. Treatment of Mycobacterium abscessus Pulmonary Disease [J]. Chest, 2022, 161(1): 64-75.

[4] van der Laan, R., Snabilie A., Obradovic M. Meeting the challenges of NTM-PD from the perspective of the organism and the disease process: innovations in drug development and delivery [J]. Respir Res, 2022, 23(1): 376.

[5] Daley, C. L., Iaccarino J. M., Lange C., et al. Treatment of Nontuberculous Mycobacterial Pulmonary Disease: An Official ATS/ERS/ESCMID/IDSA Clinical Practice Guideline [J]. Clin Infect Dis, 2020, 71(4): 905-13.

[6] Rapid communication: key changes to treatment of multidrug- and rifampicin-resistant tuberculosis (MDR/RR-TB). [Z]. Geneva; World Health Organization. 2018

[7] Ruth, M. M., Sangen J. J. N., Remmers K., et al. A bedaquiline/clofazimine combination regimen might add activity to the treatment of clinically relevant non-tuberculous mycobacteria [J]. J Antimicrob Chemother, 2019, 74(4): 935-43.

[9] Pym, A. S., Diacon A. H., Tang S. J., et al. Bedaquiline in the treatment of multidrug- and extensively drug-resistant tuberculosis [J]. Eur Respir J, 2016, 47(2): 564-74.

[10] Zhang, S. J., Yang Y., Sun W. W., et al. Effectiveness and safety of bedaquiline-containing regimens for treatment on patients with refractory RR/MDR/XDR-tuberculosis: a retrospective cohort study in East China [J]. BMC Infect Dis, 2022, 22(1): 715.

[11] Gao, M., Gao J., Xie L., et al. Early outcome and safety of bedaquiline-containing regimens for treatment of MDR- and XDR-TB in China: a multicentre study [J]. Clin Microbiol Infect, 2021, 27(4): 597-602.

[12] Huang, Z., Luo W., Xu D., et al. Discovery and preclinical profile of sudapyridine (WX-081), a novel anti-tuberculosis agent [J]. Bioorg Med Chem Lett, 2022, 71: 128824.

[13] Yao, R., Wang B., Fu L., et al. Sudapyridine (WX-081), a Novel Compound against Mycobacterium tuberculosis [J]. Microbiol Spectr, 2022, 10(1): e0247721.

[14] Zhu, R., Shang Y., Chen S., et al. In Vitro Activity of the Sudapyridine (WX-081) against Non-Tuberculous Mycobacteria Isolated in Beijing, China [J]. Microbiol Spectr, 2022, 10(6): e0137222.

[15] Brown-Elliott, B. A., Philley J. V., Griffith D. E., et al. In Vitro Susceptibility Testing of Bedaquiline against Mycobacterium avium Complex [J]. Antimicrob Agents Chemother, 2017, 61(2).

[16] Kim, D. H., Jhun B. W., Moon S. M., et al. In Vitro Activity of Bedaquiline and Delamanid against Nontuberculous Mycobacteria, Including Macrolide-Resistant Clinical Isolates [J]. Antimicrob Agents Chemother, 2019, 63(8).

[17] Pang, Y., Zheng H., Tan Y., et al. In Vitro Activity of Bedaquiline against Nontuberculous Mycobacteria in China [J]. Antimicrob Agents Chemother, 2017, 61(5).

[1] Cowman, S., van Ingen J., Griffith D. E., et al. Non-tuberculous mycobacterial pulmonary disease [J]. Eur Respir J, 2019, 54(1).

[2] Dahl, V. N., Molhave M., Floe A., et al. Global trends of pulmonary infections with nontuberculous mycobacteria: a systematic review [J]. Int J Infect Dis, 2022, 125: 120-31.

[3] Griffith, D. E., Daley C. L. Treatment of Mycobacterium abscessus Pulmonary Disease [J]. Chest, 2022, 161(1): 64-75.

[4] van der Laan, R., Snabilie A., Obradovic M. Meeting the challenges of NTM-PD from the perspective of the organism and the disease process: innovations in drug development and delivery [J]. Respir Res, 2022, 23(1): 376.

[5] Daley, C. L., Iaccarino J. M., Lange C., et al. Treatment of Nontuberculous Mycobacterial Pulmonary Disease: An Official ATS/ERS/ESCMID/IDSA Clinical Practice Guideline [J]. Clin Infect Dis, 2020, 71(4): 905-13.

[6] Rapid communication: key changes to treatment of multidrug- and rifampicin-resistant tuberculosis (MDR/RR-TB). [Z]. Geneva; World Health Organization. 2018

[7] Ruth, M. M., Sangen J. J. N., Remmers K., et al. A bedaquiline/clofazimine combination regimen might add activity to the treatment of clinically relevant non-tuberculous mycobacteria [J]. J Antimicrob Chemother, 2019, 74(4): 935-43.

[9] Pym, A. S., Diacon A. H., Tang S. J., et al. Bedaquiline in the treatment of multidrug- and extensively drug-resistant tuberculosis [J]. Eur Respir J, 2016, 47(2): 564-74.

[10] Zhang, S. J., Yang Y., Sun W. W., et al. Effectiveness and safety of bedaquiline-containing regimens for treatment on patients with refractory RR/MDR/XDR-tuberculosis: a retrospective cohort study in East China [J]. BMC Infect Dis, 2022, 22(1): 715.

[11] Gao, M., Gao J., Xie L., et al. Early outcome and safety of bedaquiline-containing regimens for treatment of MDR- and XDR-TB in China: a multicentre study [J]. Clin Microbiol Infect, 2021, 27(4): 597-602.

[12] Huang, Z., Luo W., Xu D., et al. Discovery and preclinical profile of sudapyridine (WX-081), a novel anti-tuberculosis agent [J]. Bioorg Med Chem Lett, 2022, 71: 128824.

[13] Yao, R., Wang B., Fu L., et al. Sudapyridine (WX-081), a Novel Compound against Mycobacterium tuberculosis [J]. Microbiol Spectr, 2022, 10(1): e0247721.

[14] Zhu, R., Shang Y., Chen S., et al. In Vitro Activity of the Sudapyridine (WX-081) against Non-Tuberculous Mycobacteria Isolated in Beijing, China [J]. Microbiol Spectr, 2022, 10(6): e0137222.

[15] Brown-Elliott, B. A., Philley J. V., Griffith D. E., et al. In Vitro Susceptibility Testing of Bedaquiline against Mycobacterium avium Complex [J]. Antimicrob Agents Chemother, 2017, 61(2).

[17] Pang, Y., Zheng H., Tan Y., et al. In Vitro Activity of Bedaquiline against Nontuberculous Mycobacteria in China [J]. Antimicrob Agents Chemother, 2017, 61(5).

[18] Yu, X., Gao X., Li C., et al. In Vitro Activities of Bedaquiline and Delamanid against Nontuberculous Mycobacteria Isolated in Beijing, China [J]. Antimicrob Agents Chemother, 2019, 63(8).

[1] Cowman, S., van Ingen J., Griffith D. E., et al. Non-tuberculous mycobacterial pulmonary disease [J]. Eur Respir J, 2019, 54(1).

[2] Dahl, V. N., Molhave M., Floe A., et al. Global trends of pulmonary infections with nontuberculous mycobacteria: a systematic review [J]. Int J Infect Dis, 2022, 125: 120-31.

[3] Griffith, D. E., Daley C. L. Treatment of Mycobacterium abscessus Pulmonary Disease [J]. Chest, 2022, 161(1): 64-75.

[4] van der Laan, R., Snabilie A., Obradovic M. Meeting the challenges of NTM-PD from the perspective of the organism and the disease process: innovations in drug development and delivery [J]. Respir Res, 2022, 23(1): 376.

[5] Daley, C. L., Iaccarino J. M., Lange C., et al. Treatment of Nontuberculous Mycobacterial Pulmonary Disease: An Official ATS/ERS/ESCMID/IDSA Clinical Practice Guideline [J]. Clin Infect Dis, 2020, 71(4): 905-13.

[6] Rapid communication: key changes to treatment of multidrug- and rifampicin-resistant tuberculosis (MDR/RR-TB). [Z]. Geneva; World Health Organization. 2018

[7] Ruth, M. M., Sangen J. J. N., Remmers K., et al. A bedaquiline/clofazimine combination regimen might add activity to the treatment of clinically relevant non-tuberculous mycobacteria [J]. J Antimicrob Chemother, 2019, 74(4): 935-43.

[9] Pym, A. S., Diacon A. H., Tang S. J., et al. Bedaquiline in the treatment of multidrug- and extensively drug-resistant tuberculosis [J]. Eur Respir J, 2016, 47(2): 564-74.

[10] Zhang, S. J., Yang Y., Sun W. W., et al. Effectiveness and safety of bedaquiline-containing regimens for treatment on patients with refractory RR/MDR/XDR-tuberculosis: a retrospective cohort study in East China [J]. BMC Infect Dis, 2022, 22(1): 715.

[11] Gao, M., Gao J., Xie L., et al. Early outcome and safety of bedaquiline-containing regimens for treatment of MDR- and XDR-TB in China: a multicentre study [J]. Clin Microbiol Infect, 2021, 27(4): 597-602.

[12] Huang, Z., Luo W., Xu D., et al. Discovery and preclinical profile of sudapyridine (WX-081), a novel anti-tuberculosis agent [J]. Bioorg Med Chem Lett, 2022, 71: 128824.

[13] Yao, R., Wang B., Fu L., et al. Sudapyridine (WX-081), a Novel Compound against Mycobacterium tuberculosis [J]. Microbiol Spectr, 2022, 10(1): e0247721.

[14] Zhu, R., Shang Y., Chen S., et al. In Vitro Activity of the Sudapyridine (WX-081) against Non-Tuberculous Mycobacteria Isolated in Beijing, China [J]. Microbiol Spectr, 2022, 10(6): e0137222.

[15] Brown-Elliott, B. A., Philley J. V., Griffith D. E., et al. In Vitro Susceptibility Testing of Bedaquiline against Mycobacterium avium Complex [J]. Antimicrob Agents Chemother, 2017, 61(2).

[16] Kim, D. H., Jhun B. W., Moon S. M., et al. In Vitro Activity of Bedaquiline and Delamanid against Nontuberculous Mycobacteria, Including Macrolide-Resistant Clinical Isolates [J]. Antimicrob Agents Chemother, 2019, 63(8).

[17] Pang, Y., Zheng H., Tan Y., et al. In Vitro Activity of Bedaquiline against Nontuberculous Mycobacteria in China [J]. Antimicrob Agents Chemother, 2017, 61(5).

[18] Yu, X., Gao X., Li C., et al. In Vitro Activities of Bedaquiline and Delamanid against Nontuberculous Mycobacteria Isolated in Beijing, China [J]. Antimicrob Agents Chemother, 2019, 63(8).

[2] Dahl, V. N., Molhave M., Floe A., et al. Global trends of pulmonary infections with nontuberculous mycobacteria: a systematic review [J]. Int J Infect Dis, 2022, 125: 120-31.

[3] Griffith, D. E., Daley C. L. Treatment of Mycobacterium abscessus Pulmonary Disease [J]. Chest, 2022, 161(1): 64-75.

[4] van der Laan, R., Snabilie A., Obradovic M. Meeting the challenges of NTM-PD from the perspective of the organism and the disease process: innovations in drug development and delivery [J]. Respir Res, 2022, 23(1): 376.

[5] Daley, C. L., Iaccarino J. M., Lange C., et al. Treatment of Nontuberculous Mycobacterial Pulmonary Disease: An Official ATS/ERS/ESCMID/IDSA Clinical Practice Guideline [J]. Clin Infect Dis, 2020, 71(4): 905-13.

[6] Rapid communication: key changes to treatment of multidrug- and rifampicin-resistant tuberculosis (MDR/RR-TB). [Z]. Geneva; World Health Organization. 2018

[1] Cowman, S., van Ingen J., Griffith D. E., et al. Non-tuberculous mycobacterial pulmonary disease [J]. Eur Respir J, 2019, 54(1).

[7] Ruth, M. M., Sangen J. J. N., Remmers K., et al. A bedaquiline/clofazimine combination regimen might add activity to the treatment of clinically relevant non-tuberculous mycobacteria [J]. J Antimicrob Chemother, 2019, 74(4): 935-43.

[9] Pym, A. S., Diacon A. H., Tang S. J., et al. Bedaquiline in the treatment of multidrug- and extensively drug-resistant tuberculosis [J]. Eur Respir J, 2016, 47(2): 564-74.

[10] Zhang, S. J., Yang Y., Sun W. W., et al. Effectiveness and safety of bedaquiline-containing regimens for treatment on patients with refractory RR/MDR/XDR-tuberculosis: a retrospective cohort study in East China [J]. BMC Infect Dis, 2022, 22(1): 715.

[11] Gao, M., Gao J., Xie L., et al. Early outcome and safety of bedaquiline-containing regimens for treatment of MDR- and XDR-TB in China: a multicentre study [J]. Clin Microbiol Infect, 2021, 27(4): 597-602.

[12] Huang, Z., Luo W., Xu D., et al. Discovery and preclinical profile of sudapyridine (WX-081), a novel anti-tuberculosis agent [J]. Bioorg Med Chem Lett, 2022, 71: 128824.

[13] Yao, R., Wang B., Fu L., et al. Sudapyridine (WX-081), a Novel Compound against Mycobacterium tuberculosis [J]. Microbiol Spectr, 2022, 10(1): e0247721.

[14] Zhu, R., Shang Y., Chen S., et al. In Vitro Activity of the Sudapyridine (WX-081) against Non-Tuberculous Mycobacteria Isolated in Beijing, China [J]. Microbiol Spectr, 2022, 10(6): e0137222.

[15] Brown-Elliott, B. A., Philley J. V., Griffith D. E., et al. In Vitro Susceptibility Testing of Bedaquiline against Mycobacterium avium Complex [J]. Antimicrob Agents Chemother, 2017, 61(2).

[16] Kim, D. H., Jhun B. W., Moon S. M., et al. In Vitro Activity of Bedaquiline and Delamanid against Nontuberculous Mycobacteria, Including Macrolide-Resistant Clinical Isolates [J]. Antimicrob Agents Chemother, 2019, 63(8).

[17] Pang, Y., Zheng H., Tan Y., et al. In Vitro Activity of Bedaquiline against Nontuberculous Mycobacteria in China [J]. Antimicrob Agents Chemother, 2017, 61(5).

[18] Yu, X., Gao X., Li C., et al. In Vitro Activities of Bedaquiline and Delamanid against Nontuberculous Mycobacteria Isolated in Beijing, China [J]. Antimicrob Agents Chemother, 2019, 63(8).

[20] Nie, W., Gao S., Su L., et al. Antibacterial activity of the novel compound Sudapyridine (WX-081) against Mycobacterium abscessus [J]. Front Cell Infect Microbiol, 2023, 13: 1217975.
